# Hormone receptor expression in low-grade adenosquamous carcinoma of the breast progressing to high-grade metaplastic carcinoma: A case report

**DOI:** 10.1097/MD.0000000000039131

**Published:** 2024-07-26

**Authors:** Lei Leng, Xin Hua, Wei Liu, Wu Jian

**Affiliations:** a Department of Pathology, Guangzhou Red Cross Hospital affiliated to Jinan University Guangzhou, Guangdong Province, China; b Department of Breast Surgery, Guangzhou Red Cross Hospital, Jinan University, Guangzhou, Guangdong, China; c Department of Otorhinolaryngology—Head and Neck Surgery, Guangzhou Red Cross Hospital of Jinan University, Guangzhou, Guangdong, China.

**Keywords:** breast, case report, low-grade adenosquamous carcinoma, metaplastic breast carcinoma

## Abstract

**Rationale:**

Breast low-grade adenosquamous carcinoma is an uncommon cancer that has been neglected in genetic and pathophysiological research. Consequently, medical practitioners face challenges in the effective diagnosis and treatment of this condition.

**Patient concerns:**

We present the case of a 57-year-old Asian female patient who presented with bilateral breast masses on physical examination. Ultrasound and an MRI revealed a highly suspicious malignant mass in her right breast that was completely removed surgically.

**Diagnoses:**

After pathological analysis, the diagnosis was low-grade adenosquamous carcinoma with local high-grade transformation, and some of the tumor components were estrogen receptor positive.

**Interventions:**

The patient underwent appropriate postoperative chemotherapy and achieved a favorable outcome.

**Outcomes:**

During the follow-up period after surgical resection, the patient did not experience any local recurrence or distant metastasis.

**Lessons:**

Owing to the rare combination of estrogen receptor positivity and high-grade progression, this patient also required adjuvant chemotherapy. This enhances the essential foundation for diagnosing and treating this rare disease, and facilitates the implementation of treatment plans.

## 1. Introduction

Based on an NCDB analysis spanning 2004 to 2015, 1932,688 female patients were diagnosed with invasive breast carcinoma. Among these, only 453 were identified as adenosquamous carcinoma (ASC), accounting for a prevalence rate of 0.0002%. The SEER database recorded 651,065 women with invasive breast cancer during the same period, and only 151 were classified as ASC (prevalence: 0.00027%).^[1,2]^ Metaplastic breast carcinoma (MBC) is a rare subtype of breast cancer characterized by diverse histomorphology and distinct biological features, constituting 0.2–1.0% of all invasive breast carcinomas. This poses challenges for diagnosis and has a poor prognosis. Low-grade adenosquamous carcinoma (LGASC) is a rare MBC that occurs in the breast parenchyma and has more favorable prognostic characteristics than other metaplastic carcinomas.^[3]^ Although most LGASC patients present with the triple-negative breast cancer (TNBC) subtype, isolated instances of ER-positive (estrogen receptor) patients have been documented, suggesting potential variations in treatment options.^[4,5]^

We report the case of a 57-year-old female patient with LGASC with high-grade progression and hormone receptor ER fraction positivity. To the best of our knowledge, this is the first report on such a case.

## 2. Case report

A 57-year-old female patient was found to have bilateral breast masses without any clinical symptoms during a routine physical examination. Six years ago, the patient underwent segmental surgery of her left breast and was later diagnosed with fibrocystic breast disease. The patient underwent total hysterectomy and right adnexectomy 7 years ago, which revealed leiomyoma of the uterine and right ovarian fibroma on histopathologic examination. She suffered from hypertension and had been taking levamlodipine benzenesulfonate tablets (2.5 mg) consistently for a long time, which effectively controlled her blood pressure. The patient had no personal or family history of breast cancer. On breast examination, a 1.2 cm × 1.0 cm mass with a tough texture, clear margins, and solid mobility was palpated in the outer upper quadrant of the left breast. 1.5 cm × 1.0 cm, tough, transparent, and mobile mass was palpated in the upper quadrant of the right breast. Further examination of breast masses is necessary to determine the appropriate course of action.

**Figure 1.**
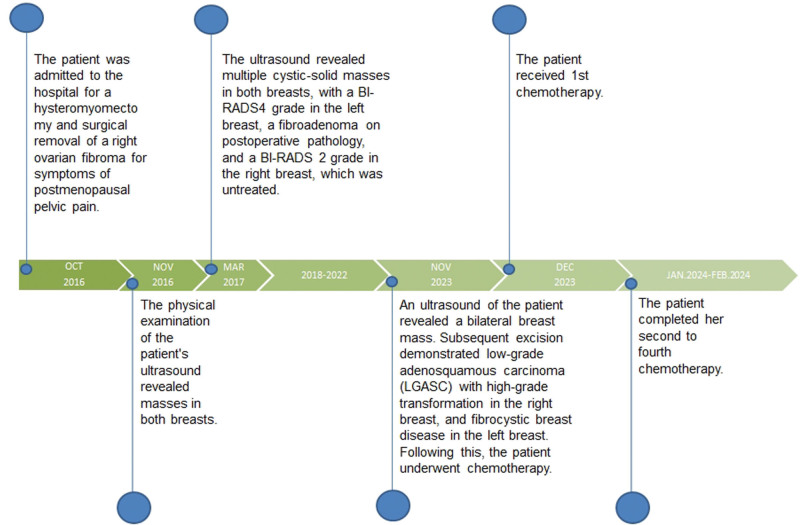
The patient’s treatment history and current information on breast-related diseases are organized on a timeline.

**Figure 2.**
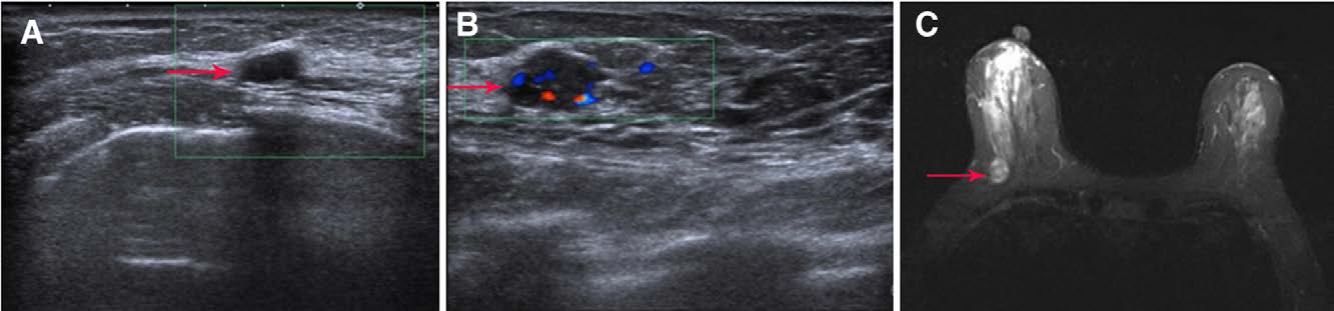
Breast imaging diagram. (A) Ultrasound image of the left breast mass. (B) Ultrasound image of the right breast nodule. (C) Bilateral breast MRI with red arrows indicating nodules in the right breast.

We reviewed the patients’ visits in chronological order (Fig. 1). Ultrasound examination revealed a spatially limited lesion of an unspecified nature in both breasts. A hypoechoic nodule with clear borders and intact margins was found at the 9 o’clock position, 20 mm from the nipple in the left breast. The surrounding tissues and structures were normal. Distinct layers with typical shapes and contours were observed in the right breast. At the 9 o’clock position, 45 mm from the nipple, a hypoechoic nodule measuring approximately 11.9 × 7.4 mm was found with an irregular shape, poorly defined margins, poorly rounded edges, and slightly increased posterior echogenicity. The surrounding tissues and structures were normal. The lesion on the right side was classified as BI-RADS IVB, indicating moderate suspicion of malignancy, whereas the entire left lesion was classified as BI-RADS IVA, indicating a benign nature (Fig. 2A,B). An MRI showed a nodular lesion in the lower outer quadrant of the right breast with a heterogeneous T2 signal (Fig. 2C). The lesion had a slightly hyperintense signal in the central region, surrounded by a double ring of slightly hyperintense to slightly hyperintense signals. A diffuse border was observed in the center, accompanied by a rim with slight irregularities. Dynamic contrast-enhanced imaging showed ring-shaped enhancement with a plateau in the temporal signal curve, indicating breast carcinoma classified as BI-RADS IVB. Additionally, a slight fibroadenoma was observed in the outer quadrant of the left breast. Therefore, surgical removal of the tumor is recommended.

During the surgery, surgeons first excised the tumor from the right breast and then the mass on the left. Macroscopically, the tumor on the right side presented as a solid mass with a pale-yellow cut surface and was accompanied by a tiny, firm nodule measuring 0.8 cm in diameter on the sectioned area and necrotic material resembling acne. The excised tissue was fixed in 10% neutral buffered formalin and processed for examination. The cells were then stained with hematoxylin and eosin and observed under a microscope.

**Figure 3.**
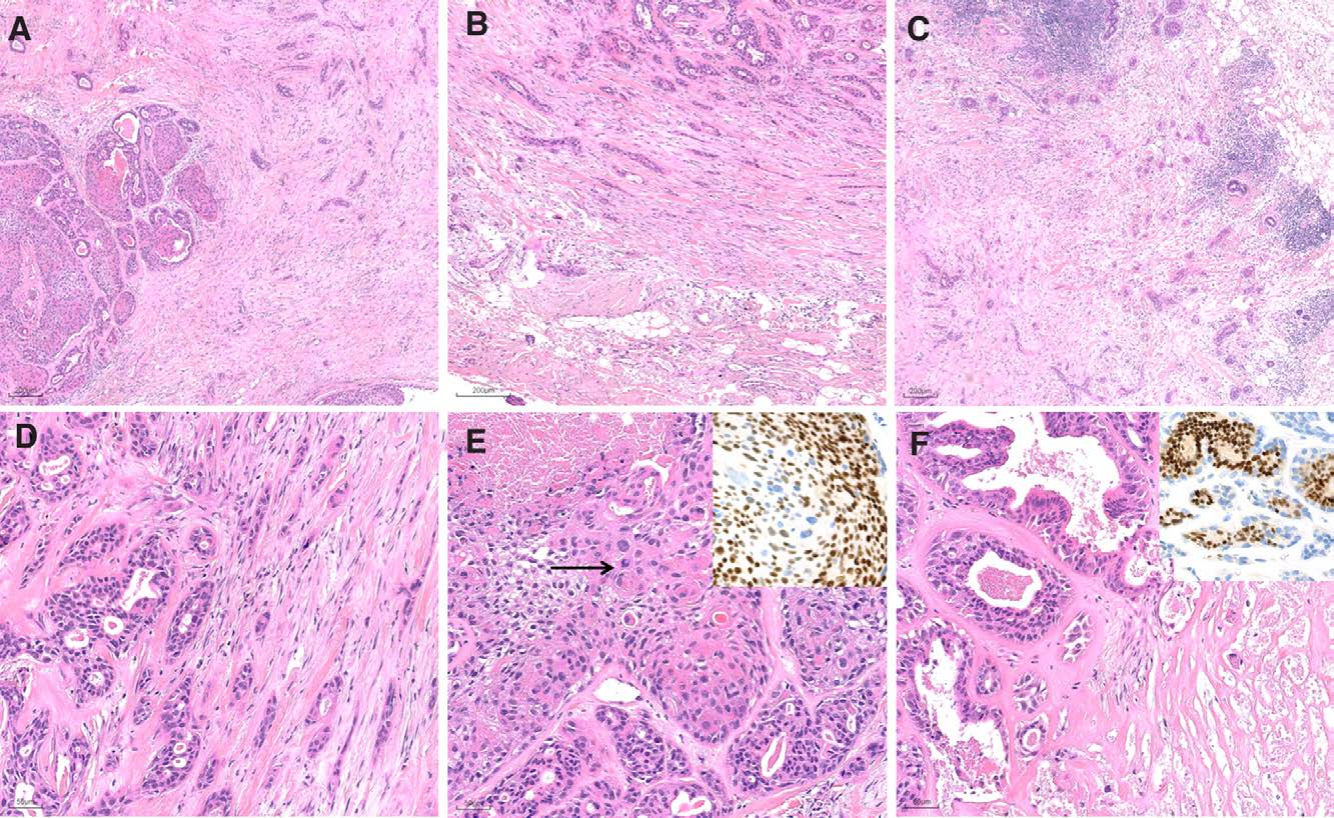
Pathological findings of LGASC. (A) nodular tumor; (B, C) hyperplasia of interstitial fibrous tissue; (C) lymphocyte infiltration; (D, E, F) striated and sieve-like pattern; necrosis and mitosis. ER (E, F) positive expression in tumor. ER = estrogen receptor, LGASC = low-grade adenosquamous carcinoma.

Microscopically, the tumor in the right breast exhibited nodular morphology with indistinct margins. It contained irregularly arranged channels and nests of solid epithelial cells embedded in fibrotic/sclerotic mesenchyme (Fig. 3A). Infiltrating ductal cell nests within the breast parenchyma displayed irregular shapes and sizes, resembling commas or tadpoles (Fig. 3B,C). The surrounding nodules were areas of extruded and deformed glandular ducts encased in sclerosis. Some of these ducts appeared striated and sieve-like (Fig. 3D–F). The glandular epithelium showed mild-to-moderate atypia, with cuboidal basaloid cells covering the outer layer of the ducts (Fig. 3E,F). Inflammatory cells were observed in most parts of the nodules (Fig. 3C). The elongated and comma-shaped glandular ducts in the outer regions of the nodule had a chaotic appearance (Fig. 3B,D). Adeno-tubular structures within the collagens interstitium appeared disorganized, with some neoplastic glands and interstitium showing necrosis and degeneration (see Fig. 3F). Additionally, there was an adjacent neoplastic squamous component of the neoplastic gland, characterized by slightly atypical hyperplastic epithelial cells displaying intracellular keratosis and pathological mitosis (Fig. 3E). Dilated ducts, intraductal papillomas, and adenosis were observed in proximity to the lesion area. Sentinel lymph node biopsy did not reveal any lymph node metastasis.

**Figure 4.**
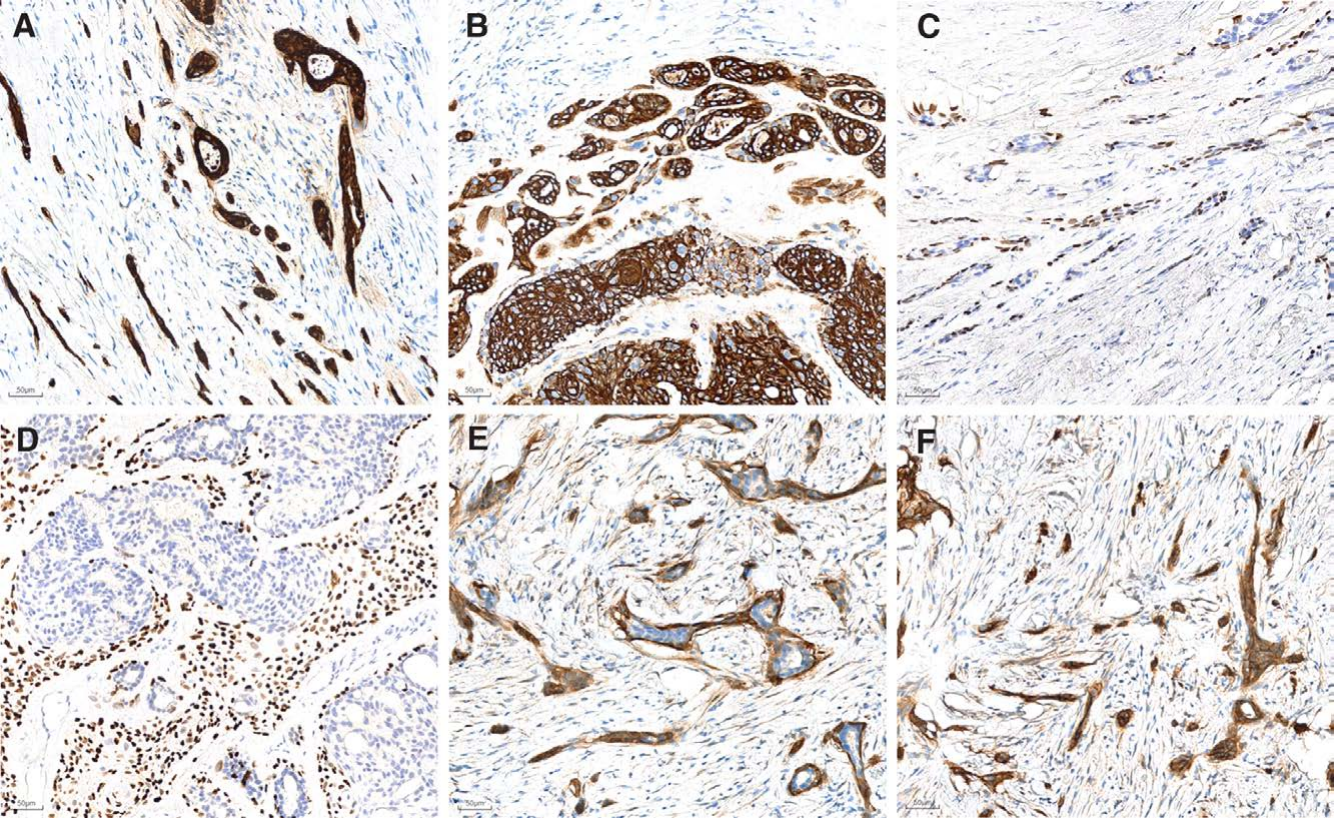
Pathological IHC findings of LGASC. CK7 (A), CK5/6 (B), and lesion tissue were strongly positive. Expression of P40 (C), P63 (D), and calponin (E) is observed around lesional glands. (F) Positive staining for EGFR. CK7 = cytokeratin 7, IHC = immunohistochemical, LGASC = low-grade adenosquamous carcinoma.

To accurately describe the tumor components, we performed immunohistochemical (IHC) staining of a representative paraffin block section using the ChemMate Envision method (DakoCytomation, Glostrup, Denmark). Our analysis of LGASC revealed focal ER positivity (Fig. 3E,F), PR (progesterone receptor) negativity, HER2 (human epidermal growth factor receptor 2) negativity, and CK7 positivity (cytokeratin; Fig. 4A). In comparison to its robust expression in LGASC (Fig. 4A), CK5/6 positivity was patchy and insufficient in sclerosing adenopathy of the breast and intraductal papillomas (Fig. 4B). P40, P63, and calponin were expressed positively at the periphery of the tumor nests and resembled myoepithelial cells (Fig. 4C–E). Both the squamous components of the tumor and periglandular tumor cells were positive for P63. The tumor was positive for epidermal growth factor receptor (EGFR; Fig. 4F). It has a low overall proliferation rate, with Ki-67 positivity ranging from 2% to 10%. Our reported cases and review of the relevant literature indicate that not all tumor components of LGASC are triple-negative. In cases which patients are hormone receptor-positive, appropriate chemotherapy may be considered after surgical resection. Moreover, histopathological examination following surgical removal of the left breast mass confirmed the diagnosis of fibrocystic breast disease.

The patient underwent simple right mastectomy and sentinel lymph node biopsy. Pathological examination revealed a cancerous nodule with a maximum diameter of approximately 0.8 cm; no lymph node metastases were detected, and she was diagnosed with LGASC (pT1N0M0) with high-grade progression. A course of chemotherapy with docetaxel 120 mg and cyclophosphamide 1000 mg for 4 months was then administered. The patient did not experience any significant discomfort during chemotherapy.

## 3. Discussion

LGASC is a rare form of breast cancer first described by Rosen and Ernsberger in 1987.^[6]^ Stuart et al reviewed 155 published cases,^[7]^ while Lewis analyzed 25 cases in the literature,^[3]^ with several additional case reports and 1 case of LGASC in men.^[3, 7, 8]^ The age of the original LGASC study population ranged from 42 to 76 years, with an average age of 59 years.^[6]^ Subsequent studies have shown that the age of onset ranges from 19 to 88 years, with the disease being more common in menopausal women. Unlike other breast cancer subtypes, LGASC has no typical imaging or clinical features and can occur anywhere in the breast parenchyma.^[9]^ The most common clinical presentation of LGASC is a palpable mass accompanied by abnormal mammogram or nipple discharge. The rarity of cases also contributes to a lack of knowledge of LGASC.

Regarding the pathomorphology of LGASC of the breast, the 3 components comprise the background of glandular or tubular structures, squamous epithelium, or reactive connective tissue hyperplasia, with the glandular or tubular structures being closely mixed with the squamous epithelium or having an overabundance of them. The glandular or tubular structures were small and rounded, with little cellular heterogeneity and few mitotic cells. The infiltrating glandular tubular structures showed myoepithelial differentiation around them. Squamous epithelial components may be solidly striated, keratinized beads, or keratinized cysts. Marked proconnective tissue proliferation was observed in the interstitium, which formed a spindle cell background. Foci of peripheral lymphocytic infiltration are often observed.^[10]^ High-grade adenosquamous carcinoma transformation is suggested when some of the tumors show moderate to high cellularity, and heterogeneity, or neoplastic necrosis. Van Hoeven et al observed 11 cases of LGASC, characterized by local infiltration and a tendency to metastasize after multiple recurrences or high-grade transformations, typically when the tumor was larger than 3 cm.^[4]^

Immunohistochemical staining for P40, P63, and calponin was positive, suggesting that the tumor originated from the primitive myoepithelium of the mammary gland. Positive staining for EGFR and P63 suggests that the tumor possesses the characteristics of basal-like breast carcinoma. Additionally, strong positivity for CK7 and CK5/6 suggests that the tumor possesses the characteristics of adenocarcinoma and squamous carcinoma in the direction of differentiation. Furthermore, mild expression of the ER receptor was observed in this case.^[11]^

Diagnosing LGASC solely on the basis of its characteristic histopathology is difficult because of the complexity of its immunophenotypes and specific changes. LGASC is often accompanied by sclerosing adenosis, intraductal papillomas, and other lesions. While intact myoepithelial staining is observed in sclerosing adenosis, LGASC shows a characteristic range of changes in the myoepithelial cells surrounding the tumor nests, ranging from intact to partially absent or completely absent. In contrast to LGASC, syringomatous adenomas of the nipple occur mainly in the nipple or areola and not in the parenchyma. Adenosquamous proliferation (ASP) and LGASC can be difficult to distinguish because of their similar histological morphologies and immunophenotypes. However, LGASC lesions are typically more extensive than ASP lesions. Moreover, the presence of fibrous tissue at the margins of the lesion or infiltrative growth between the normal breast lobules may be evidence of LGASC.^[12]^ Compared to other types of breast cancer, the ductal tumor component of LGASC is highly differentiated and often indistinguishable from non-tumorous glands. ASC of the breast is a combination of squamous cell carcinoma and adenocarcinoma, with varying degrees of differentiation of the squamous epithelial component, whereas adenocarcinoma commonly presents as an invasive ductal carcinoma in the absence of the periductal myoepithelium.^[3,9]^

LGASC is regularly positive for P63 and negative for ER, PR, and HER2, and shares the molecular pathophysiology, gene expression profile, and immunophenotype of “triple-negative” and complicated basal subtypes of breast cancer.^[11]^ It was found that the most significant cancer-associated alterations in TNBC patients were TP53 mutations (74%), followed by PIK3CA (18%), KMT2C (7%), and PTEN mutations (6%). The PIK3CA mutation rate is higher in Chinese TNBC patients, and the genomic characteristics of BLIS-TNBC with increased homologous recombination disorder (HRD) and BLIS-TNBC with low HRD are different.^[13]^ LGASC has a high rate of PIK3CA mutations and amplification of the epidermal growth factor receptor, but no TP53 mutations.^[14]^ The 2 other cases of LGASC and associated MSC carry the GNASc.C2530T: p. Arg844Cys mutation, which may promote the development of advanced metaplastic carcinoma of the breast.^[15]^ In addition, 1 patient was a carrier of the BRCA1 gene.^[16]^ TNBC is extremely heterogeneous and lacks molecular targets for its treatment. It is generally recognized that both triple-negative and basal-like carcinomas exhibit more aggressive clinical manifestations.^[11]^ Therefore, the inclusion of HRD in expression-based BLIS subtypes may show that patients with high HRD benefit from chemotherapy, whereas those with low HRD have no specific treatment options and should be considered for participation in clinical trials. As far as the prognosis is concerned, LGASC is a type of low-malignant anaplastic carcinoma. This means that in most cases, no recurrence occurs after total mastectomy, whereas in some cases, recurrence may occur after simple excision of the mass. Only a few cases of metastasis to the lungs or axillary lymph nodes or even death have been reported. However, the efficacy of radiotherapy and chemotherapy for LGASC remains unclear.^[13]^

In previous studies, surgical excisional biopsy was performed in 67% of low-grade adenosquamous breast cancer cases, while mastectomy was performed in 33% of cases. Recent studies have shown that LGASC has a low rate of local recurrence without nodal or distant metastases; however, the optimal treatment has not yet been determined. However, the benefits of adjuvant radiotherapy have not yet been studied. Compared with other triple-negative MBCs, which have a poor prognosis and require radiotherapy and chemotherapy after resection, chemotherapy does not appear to be necessary for triple-negative LGASC, and hormone therapy is not recommended. Radiotherapy is required when a conservative approach is used.^[11]^ In this study, we present a rare case of partially ER-positive LGASC with high-grade transformation. Postoperative adjuvant chemotherapy differed significantly from the conservative treatment approach for triple-negative LGASC. Throughout the follow-up period, no evidence of tumor recurrence or metastasis was observed. Considering the infrequency of hormone receptor-positive LGASC cases, we will prolong the patient follow-up period to observe the efficacy of treatment.

## 4. Conclusion

This case presents a rare occurrence of LGASC in the breast, wherein resection and pathological examination revealed the coexistence of partially ER-positive cells and high-grade tumor transformation. In summary, this case enhances our understanding of this uncommon disease and broadens available treatment options.

## Acknowledgments

First, we would like to express our sincere appreciation to the patient who participated in this study and generously granted us permission to share her medical information. Her invaluable cooperation and willingness to participate in this study are highly acknowledged. We extend our deepest gratitude to all the individuals who contributed to the completion of this medical case report. The final manuscript has been approved by all authors who have collectively agreed to assume responsibility for all aspects of this work.

## Author contributions

**Funding acquisition:** Xin Hua, Wei Liu.

**Writing – original draft:** Lei Leng, Wu Jian.

**Writing – review & editing:** Lei Leng, Xin Hua, Wei Liu.
